# The Influence, Barriers to and Facilitators of Anterior Cruciate Ligament Rehabilitation Adherence and Participation: a Scoping Review

**DOI:** 10.1186/s40798-020-00258-7

**Published:** 2020-07-17

**Authors:** Adam Walker, Wayne Hing, Anna Lorimer

**Affiliations:** 1 Faculty of Health Sciences and Medicine, Bond University, Gold Coast, 4226 Australia; 2 Bond Institute of Health and Sport, Promethean Way, Robina, QLD 4226 Australia

**Keywords:** Anterior cruciate ligament, Physiotherapy, Return to sport, Adherence, Compliance, Rehabilitation

## Abstract

**Background:**

Outcomes following anterior cruciate ligament (ACL) reconstruction are considered poor. There are many factors which may influence patient outcomes. As such, the purpose of this review was to report on the influence, barriers to and facilitators of rehabilitation adherence and participation after ACL reconstruction, providing information to help clinicians and patients make quality decisions to facilitate successful rehabilitation.

**Methods:**

A systematic search of five electronic databases was undertaken in identifying studies from inception to 18 July 2019. The search included English language articles reporting on the influence, barriers to and facilitators of adherence and participation in rehabilitation of patients who have undergone ACL reconstruction. Data extraction and synthesis of included studies were undertaken.

**Results:**

Full text articles (*n* = 180) were assessed for eligibility following screening of titles and abstracts (*n* = 1967), yielding 71 studies for inclusion. Forty-four articles investigated ‘rehabilitation prescription and participation’ and 36 articles investigated ‘rehabilitation barriers and facilitators’. The results indicate that a moderately or minimally supervised rehabilitation program is at least as effective as a fully supervised high-frequency rehabilitation program, although a longer duration of supervised rehabilitation is associated with improvement in a multitude of functional outcomes. A number of psychological factors associated with rehabilitation adherence were also identified. The most commonly investigated concepts were self-motivation, athletic identity and social support. Patients perceived the therapeutic relationship, interaction with family and friends, self-motivation, fear of reinjury, organisation/lack of time and interpersonal comparison as the most common barriers to and facilitators of rehabilitation.

**Conclusions:**

A longer duration of supervised rehabilitation is associated with an increased chance of meeting functional and return to sport criteria; however, the optimal supervised rehabilitation frequency is yet to be determined. Identification of the barriers to and facilitators of adherence and participation in ACL rehabilitation provides an opportunity for further research to be conducted to address personal, environmental and treatment-related factors, with the aim to improve rehabilitation outcomes.

## Key Points

- A longer duration of supervised rehabilitation is associated with more favourable post-operative outcomes.
- The optimal frequency of supervised post-operative rehabilitation is unknown.
- Patients experience a variety of psychological, environmental, personal and treatment-related barriers to and facilitators of rehabilitation.

## Background

Anterior cruciate ligament (ACL) injury occurs during rapid valgus loading and internal tibial rotation of the knee [1]. Every year, 3% of amateur athletes injure their ACL, often requiring subsequent reconstruction [2]. Injury of the ACL is also one of the most devastating, resulting in significant time loss from sport [2], long-term functional knee impairments [3], reduced quality of life [4], financial burden [5] and early-onset osteoarthritis [6].

Despite significant advances in surgical technique, the outcomes following ACL reconstruction continue to be reported as poor [7]. Research demonstrates that only 55% of patients who undergo ACL reconstruction make a return to competitive sport [8], and between 15 and 23% of young athletes will suffer a re-rupture or injure the contralateral knee [9]. Reinjury rates are even higher for those under 18 at 33% [10].

Potentially, the underutilisation of rehabilitation in recovery from ACL injury is contributing to the poor outcomes [11]. Growing evidence suggests that due to inadequacies in current rehabilitation programs, patients return to sport (RTS) too early and with significant deficits in knee function, risking reinjury and long-term impairments [12]. There has been substantial research attempting to formulate an evidence base of what best practice ACL rehabilitation programs should include [13]. Despite this, Van Melick et al. (2016) highlighted the current lack of evidence regarding the optimal rehabilitation period or how many appointments work best for RTS [14]. Furthermore, it appears warranted to consider the contextual and personal factors of rehabilitation programs that may act as barriers to or facilitators of rehabilitation. Increased awareness and understanding of these factors may offer new insights and opportunities to improve long-term ACL reconstruction outcomes and enhance clinicians’ ability to provide patient-centred care [15].

Clinicians are therefore continuing to seek guidance on the best way to structure and deliver rehabilitation to facilitate return to sport and minimise the risk of reinjury. With that in mind, this scoping review aims:

- To report on the influence of rehabilitation adherence and participation on outcomes after ACL reconstruction
- To report on the barriers to and facilitators of adherence and participation in ACL rehabilitation
- To provide information to help clinicians and patients make quality decisions to facilitate adherence and appropriate participation in ACL rehabilitation

## Methods

A scoping review was conducted to synthesise evidence on ACL reconstruction rehabilitation adherence and participation for the clinician providing rehabilitation services to patients who have undergone ACL reconstruction. Due to the broad exploratory nature of the topic, a scoping review design and methodology was used to facilitate collation and mapping of evidence for the identification of key concepts, knowledge gaps and the types of evidence currently available [16].

### Research Questions

The research questions are:

1. What is the reported influence of adherence and participation in ACL reconstruction rehabilitation on patient outcomes?
2. Which factors are reported to influence adherence and participation in ACL reconstruction rehabilitation?

### Protocol

A single researcher (AW) conducted the literature search to identify, screen and select studies to be included in accordance with the Preferred Reporting Items for Systematic Reviews and Meta-Analysis Extension for Scoping Reviews (PRISMA-ScR) [17]. An a priori protocol was developed and published on the Open Science Framework (https://osf.io/a7tz8/?view_only=9bc5d21c0c034f70a37202abab7330c0) prior to data extraction, on the 10 August 2019. No changes were made to the protocol from publication through to completion.

### Study Design

The search strategy was developed through the application of the methodological frameworks proposed by Arksey and O’Malley (2005) [16] and Peters et al. (2015) [18]. We followed a 3-step approach:

1. A pilot search of PubMed and Embase using the medical subject headings ‘anterior cruciate ligament’ AND reconstruction AND rehabilitation AND ‘patient compliance’ (May 2019).
2. Identified keywords and terms relating to anterior cruciate ligament reconstruction rehabilitation adherence and participation (May 2019).
3. Execution of the final search strategy and further searching of reference lists of the selected articles, systematic reviews and narrative reviews (July 2019).

**Fig. 1:**
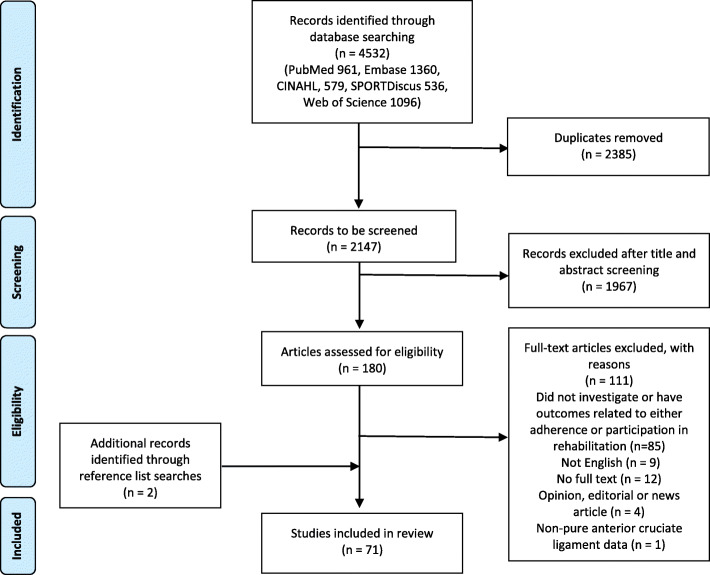
Preferred Reporting Items for Systematic Reviews and Meta-Analysis Extension for Scoping Reviews (PRISMA-SCR) flow diagram

A search was formulated (supplementary file 1) and conducted in 5 databases (PubMed, Embase, CINAHL, SPORTDiscus and Web of Science) from inception to 18 July 2019. Articles were downloaded to the EndNote reference management software (https://www.endnote.com/) for selection by AW according to the PRISMA-ScR statement [17] (Fig. 1).

#### Eligibility criteria

The eligibility criteria were defined by the *Population* (any individual that had undergone an anterior cruciate ligament reconstruction regardless of graft type or concomitant injury), *Concept* (any study reporting on the effect of adherence and participation in rehabilitation or a rehabilitation program) and *Context* (all periods of time, outcomes, comparators, follow up, rehabilitation setting and duration and type of intervention). The following types of publications were eligible for inclusion: original research, reviews, scoping reviews, systematic reviews, meta-analysis, case series and clinical commentaries.

Exclusion criteria were (a) non-English language, (b) examined pre-operative interventions or non-operative rehabilitation intervention for ACL rupture and (c) no access to the full text. The following were also excluded from our review: conference abstracts/proceedings, opinion pieces, guidelines, magazine and newspaper articles and rehabilitation protocols.

#### Data extraction

AW extracted data from publications meeting inclusion criteria into a custom Excel spreadsheet. Data extraction, categorisation and mapping were performed as per Peters et al. (2015) in an iterative process as the reviewer became more familiar with the evidence [18].

#### Synthesis and risk of bias

To answer the research questions, data were narratively synthesised by the author-defined categories: (A) rehabilitation prescription and participation and (B) barriers to and facilitators of rehabilitation. Studies in category A were further categorised into 3 sub-categories: ‘supervised rehabilitation frequency’, ‘supervised rehabilitation duration’ and ‘rehabilitation adherence’. Studies in category B were further categorised into three sub-categories: ‘psychological’, ‘patient perspectives’ and ‘other factors’. Studies may be allocated to multiple groups. Results were mapped based on the population profile (age, sex, activity level), study design and concepts investigated. The synthesis of qualitative data was guided by the methodological framework presented by Thomas and Harden (2008) [19]. In line with the recommended scoping review methodology, a quality appraisal is not required [16, 18].

## Results

The search strategy yielded 4532 citations with two additional records added following reference list searching [20, 21]. Duplicates (*n* = 2385) were removed, and exclusion based on screening of title and abstract (*n* = 1967) left 180 full-text articles which were retrieved and assessed for eligibility. Of these, 111 were excluded for the following reasons: 85 studies did not investigate or have outcomes related to either adherence or participation in rehabilitation; nine were of non-English language; 12 had no access to full text; four were opinion, editorial or news articles; and one article with a non-pure ACL data set (participants from multiple injuries). Seventy-one publications fulfilled the criteria and were included in the review (Fig. 1).

The 71 included articles included 19 reviews and 52 studies. The articles were categorised as per Table 1, with four reviews [13, 58–60] and five studies [30, 61, 62, 70, 71] classified in multiple categories. As outlined in the synthesis and risk of bias methods section, the articles in category (A) were selected to answer the first research question: What is the reported influence of adherence and participation in ACL reconstruction rehabilitation on patient outcomes? Forty-four articles consisting of 12 reviews and 32 original studies were included in this category. An article was categorised into ‘supervised rehabilitation frequency’ if it investigated the difference in outcomes between varying rates of attendance to a rehabilitation service. Most of these articles were developed to investigate home versus clinic-based rehabilitation. An article was categorised as ‘supervised rehabilitation duration’ if it investigated the association between a shorter versus longer duration of supervised rehabilitation on outcome and an article was categorised as ‘rehabilitation adherence’ if it utilised an adherence measure to determine the correlation between adherence to a prescribed rehabilitation protocol and outcome.

**Table 1:** Number of articles included in each category

	Reviews	References	Original studies	References
Category (A) Rehabilitation prescription and participation				
Supervised rehabilitation frequency	9	[13, 22–29]	20	[30–49]
Supervised rehabilitation duration	0		8	[50–57]
Rehabilitation adherence	3	[58–60]	4	[61–64]
Category (B) Rehabilitation barriers and facilitators				
Psychological	10	[13, 21, 58–60, 65–69]	8	[62, 70–76]
Patient perspectives	0		15	[15, 20, 30, 61, 71, 77–86]
Other factors	0		3	[30, 70, 87]

Bold text highlights the two main categories

The articles in category (B) were selected to answer the second research question: Which factors are reported to influence adherence and participation in ACL reconstruction rehabilitation? Thirty-six articles consisting of 10 reviews and 26 original studies were included in this category. An article was categorised into ‘psychological’ if it investigated the association between a psychological variable and adherence to rehabilitation, as ‘patient perspectives’ if the study included a qualitative research methodology reporting on patients' opinions and perspectives on barriers to and facilitators of rehabilitation and as ‘other factors’ if it did not fit the first two categories.

**Fig. 2:**
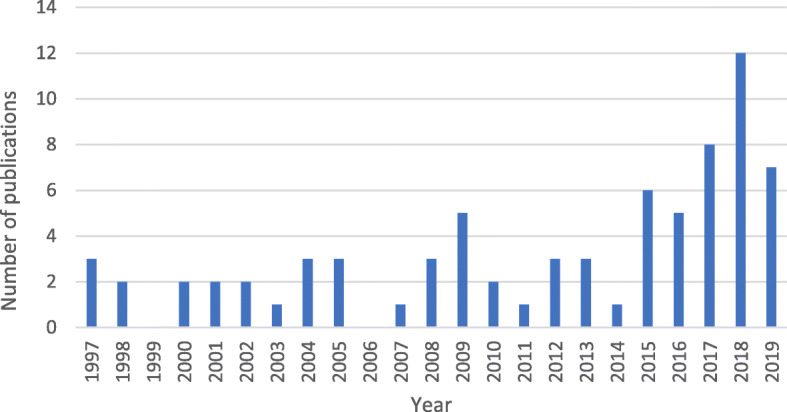
Number of academic publications by year meeting selection criteria

Publication dates varied from 1997 to 2019. The number of articles published increased substantially from 2015 (Fig. 2), illustrating the rise in interest in the topic.

## Category (A) Rehabilitation Prescription and Participation

### Supervised Rehabilitation Frequency

#### Reviews

All nine included reviews investigated home versus clinic-based or supervised rehabilitation (Table 2). Conclusions were consistent across the reviews. All systematic reviews either stated that home-based rehabilitation is as equally effective as clinic-based rehabilitation, or the evidence was inconclusive. The methodological quality assessments in each review consistently highlighted issues with the quality of the current available evidence. Anderson et al. (2016) performed the most recent review inclusive of evidence until 2014, raising questions about the currency of the evidence presented [13].

**Table 2:** Summary of included reviews investigating ‘supervised rehabilitation frequency’

Author (year)	Review type	Dates	Methodological quality	No. included original studies	Conclusion
Anderson et al. (2016) [13]	Systematic	2004-14	Not assessed	4	Inconclusive
Andersson et al. (2009) [22]	Systematic	1995-2009	Severely limited by methodology quality	7	Inconclusive
Coppola and Collins (2009) [23]	Systematic	1980-2007	Moderate quality	3	Inconclusive
Kruse et al. (2012) [24]	Systematic	2006-10	Large biases in studies	6	Equally effective
Lobb et al. (2012) [29]	Non-systematic	Until 2011	Moderate evidence	2	Equally effective
Papalia et al. (2013) [25]	Systematic	Until 2013	Good quality	10	Equally effective
Risberg et al. (2004) [26]	Systematic	Until 2003	Significant limitations across studies	3	Equally effective
Trees et al. (2005) [27]	Systematic	Until 2005	Poor	3	Equally effective
Wright et al. (2008) [28]	Systematic	Until 2005	Biases present	4	Equally effective

Methodological quality refers to the outcome of the quality appraisal undertaken by the review not the authors of this study. The conclusion stated is that of the included review in reference to the comparison of home versus clinic-based rehabilitation. The number of original studies is only those included in each review for the evaluation of home versus supervised rehabilitation

#### Original Studies

Of the 20 included original studies regarding supervised rehabilitation frequency, eight studies were retrospective in design, seven randomised controlled trials (RCT), three prospective, one cross-sectional and one case report (Table 3). The mean age was 27.6 (21.4–35.5). All but one study had male and female participants. Thirteen studies utilised the bone-patella tendon-bone (BPTB) graft, ten hamstring graft, two allografts and one did not state. Participant activity level was not stated in 13 studies, while athletes of various levels were involved in seven studies.

**Table 3:** Summary of included original studies investigating ‘supervised rehabilitation frequency’

Author (year)	Design	Population					Groups: av no. appointments (av/week)	Supervision category	Intervention period (months)	OM assessment (months)	OMs
		*n*	Age (mean)	Sex	Graft	Activity level					
Beard and Dodd (1998) [31]	RCT	31	28	M 21 F 5	BPTB	Not stated	Home: not specified Clinic + knee class: 16 (1.33)	Unspecified High	Mid (approx. 1-4)	Pre-operative, 3 and 6	Lysholm knee score, IKDC knee evaluation form, Modified Tegner activity score, VAS sports frequency and level, VAS ADLs, Isokinetic Fl and ext (60deg/sec), KT-1000
Christensen et al. (2018) [32]	Retrospective	332	31.2	M 192 F 140	HS / BPTB	Not stated	Unsupervised: 3–7 (0.16) Semi-supervised: 8–13 (0.33) Supervised: 14–35 (0.76)	Low Mod High	Late (0-8)	8	KOS-ADL, NPRS
Darian et al. (2015) [33]	Case report	1	22	M	HS	Competitive sport	Supervised: 15 (0.63)	High	Mid (0-6)	Pre-operative, 1.5, 3 and 6	IKDC subjective knee evaluation, Lysholm knee score, KOOS, K-SES, single hop
De Carlo and Sell (1997) [34]	Retrospective	180	26.8	M 130 F 50	BPTB	Sport participation (100%)	Home: 7 (0.29) Standard routine: 20 (0.83)	Low High	Mid (0-6)	1,6 and 12	Isokinetic Fl and ext (180deg/s) ROM, Modified Noyes Questionnaire
Dempsey et al. (2019) [30]	Cross-sectional	60	29.7	M 31 F 29	HS/BPTB	Competitive (57%) Recreational (41%) Non-athletes (3%)	Cohort: 58.2 (2.24)	High	Mid (av. 6.3)	At physician clearance (6.3)	Isokinetic ext (60deg/sec), isometric ext (90deg), single hop for distance, IKDC 2000 subjective knee evaluation, physiotherapy questionnaire
Feller et al. (2004) [35]	Retrospective	20	28	M 16 F 4	HS/BPTB	Not stated	Minimal: 0–3 (0.04) Intermediate: 4–11 (0.14) Regular: 15-50 (0.51)	Low Low High	Late (0–12)	12	Cincinnati knee score, Cincinnati occupational rating scale, Cincinnati sports activity level, IKDC knee evaluation form, KT-1000
Fischer at al. (1998) [36]	Prospective	54	30.5	M 28 F 26	BPTB/allograft	Not stated	Home: 5 (0.21) Clinic: 19.9 (0.83)	Low High	Mid (0–6)	Examination at 1 week, 1.5, 3, 4.5 and 6 Lysholm at 3 and 6 Hops and HSQ at 6	Lysholm knee score, HSQ, ROM, KT-1000, Effusion, PFJ crepitus, anterior draw, medial-lateral laxity, posterior draw, Lachman’s, pivot shift, thigh atrophy, single hop for distance, 6m timed hop, triple hop for distance, triple crossover hop for distance
Grant and Mohtadi (2005) [37]	RCT	145	29.3	M 85 F 60	Not stated	Not stated	Home: 3 (0.25) Clinic: 14 (1.17)	Mod High	Early (0–3)	Pre-operative, 1.5 and 3	ACL-QoL questionnaire, ROM, KT-2000, knee ROM in gait, isokinetic Fl and ext (180deg/sec)
Grant et al. (2010) [38]	RCT	88	33.7	M 58% F 42%	Not stated	Not stated	Home: 3 (0.25) Clinic: 14 (1.17)	Mod High	Early (0–3)	26-53	ACL-QoL, ROM, KT-1000, IKDC knee evaluation form, isokinetic Fl and ext (60deg/sec)
Han et al. (2015) [39]	Retrospective	93	23	M 82 F 11	HS	Recreational athletes	Non-compliant: 3.6 (0.1) Mod compliant: 10.7 (0.3) Fully compliant: 17 (0.47)	Low Mod Mod	Late (0–9)	Pre-operative 9	Self-report RTS, Cincinnati sport activity level, Lysholm knee score, KOOS, SF-36, PCS, MCS
Hohmann et al. (2011) [40]	RCT	40	28.5	M 20 F 20	BPTB	Physically active	Home: 5 (0.10) Clinic: 45 (0.86)	Low High	Late (0–12)	1.5, 3, 6, 9 and 12	Lysholm knee score, Tegner activity level scale, single hop for distance 6 m timed hop, vertical jump, isokinetic F and ext (120deg/sec), isometric ext (30deg)
Inacio et al. (2016) [41]	Retrospective	6385	28	M 4657 F 1728	HS/BPTB/allograft	Not stated	Medical encounters in days 1–90 and 91–180	N/A	Mid (0–6)	Mean 2.8 ± 1.8 years	Revisions within 6 months
Lim et al. (2019) [42]	Prospective	26	35.5	M 18 F 8	HS	Not stated	Home-based: 0 (0) Supervised: 48 (2)	Low High	Mid (0–6)	Pre-operative and 6	Isokinetic Fl and ext (60deg/sec and 180 deg/sec), Biodex stability system SD OSI
Miller et al. (2017) [43]	Retrospective	660	29.4	M 389 F 271	Not stated	Not stated	Minimal: < 9 Moderate: 9–14 High: > 15	Unable to determine	Mid (3+)	Admission and last formal PT visit	KOS-ADL, NPRS, age, sex, intervention charges, ACL revision rate, area deprivation index, payer mix
Przybylak et al. (2018) [44]	Prospective	50	30.5	M 37 F 13	HS/BPTB	Recreational athletes	Home: 6 (0.12) Supervised: 48 (0.92)	Low High	Late (0–12)	Pre-operative and 12	Kujal’s Scoring questionnaire, Tegner activity scale, KOOS, ROM, FMS
Revenas et al. (2009) [45]	RCT	51	22.5	M 33 F 18	HS / BPTB	Not stated	Guided therapy: 3 (0.07) Knee class: 15 (0.33)	Low Mod	Late (1.5–12)	Pre-operative 6 and 12	IKDC knee evaluation form, Lysholm knee score, Tegner activity level scale, isometric ext (90deg), single hop for distance, ROM
Schenck et al. (1997) [46]	RCT	37	23.5	M 28 F 9	BPTB	Not stated	Home: 2.85 (0.12)Clinic: 14.2 (0.6)	Low High	Mid (0–6)	Pre-operative, 3 and 12	ROM, Lysholm knee score, VAS pain, single hop for distance, KT-1000, SIP, level of activity, surgery satisfaction
Tracey et al. (1997) [47]	Retrospective	39	25.2	M 54 F 15	BPTB	Competitive recreational athletes (80%)	Home: 7.3 (0.30) Minimally compliant subgroup: 12 (0.5) Clinic: 60 (2.5) Non-compliant subgroup: 1.7 (0.07)	Mod Mod High Low	Mid (0–6)	16 (12–30)	ROM, anterior draw, Lachman's, pivot shift, Lysholm knee score, activity level (4 levels), surgery satisfaction
Ugutmen et al. (2008) [48]	RCT	104	31.5	M 103 F 1	HS	Not stated	Home: unspecified Clinic: unspecified	Unspecified	0–8	Mean 21.1 (12–66)	Xray, MRI, subjective comments from patients, thigh atrophy NPRS, effusion, ROM, KT-1000 PFJ pain and crepitation, Lachman's, pivot shift, anterior drawer, Lysholm knee score, HSS, IKDC knee evaluation form
Yu et al. (2017) [49]	Retrospective	42	21.4	M 39 F 3	BPTB	Not stated	Rupture: 18 (0.35) No rupture: 18.6 (0.36)	Mod Mod	0–12	N/A	Re-rupture

*ACL* anterior cruciate ligament, *ADLs* activities daily living, *BPTB* bone-patella-tendon-bone, *deg* degree, *ext* extension, *F* female, *Fl* flexion, *FMS* functional movement screen, *HS* hamstring, *HSQ* Health status questionnaire, *HSS* hospital of special surgery, *IKDC* International knee documentation committee, *KOOS* knee injury and osteoarthritis outcome score, *KOS*-*ADL* knee outcome survey–activities of daily living, *K*-*SES* knee–self-efficacy scale, *m* metre, *M* male, *MCS* mental component summary, *MRI* magnetic resonance image, *NPRS* numeric pain rating scale, *OSI* overall stability index, *PCS* physical component summary, *PFJ* patellofemoral joint, *RCT* randomised controlled trial, *ROM* range of motion, *RTS* return to sport, *sec* seconds, *SF*-*36* short form-36, *SIP* sickness impact profile, *VAS* visual analog scale

A variety of outcome measures (OMs) were used (Table 3) with no single OM used consistently across the majority of studies. Outcome measure use was investigated by grouping the type of OM into the following categories: hop tests, isokinetic dynamometry, patient reported OMs, clinical-based OMs and other OMs. The number of studies in each category were as follows; five studies used at least one hop test [30, 33, 36, 40, 46], eight studies used an isokinetic dynamometry strength measure [30, 31, 34, 37, 38, 40, 42, 45] and 18 different patient-reported OMs were utilised in 12 different studies [30, 32–39, 43, 44, 46]. Eighteen different clinical-based assessments (pain, range of motion, atrophy, effusion, laxity, Lysholm knee score, Tegner activity scale and international knee documentation committee knee evaluation) were used in 15 studies [31–38, 40, 43–48] and 14 other OMs (RTS status/activity level, re-rupture, gait analysis, functional movement screen, surgery satisfaction, imaging and demographics) were utilised in 10 studies [38, 39, 41–44, 46–49].

To determine the correlation between frequency of supervised rehabilitation and rehabilitation outcome, the frequency of appointments in each intervention group was mapped based on the average number of weekly appointments across the intervention period. Patients who attended less than once per month were classified as low, between 1 and 2×/month as moderate and more than twice per month as high across the duration of their rehabilitation.

The intervention period was labelled according to the stages of rehabilitation the intervention spanned; early-stage (0–3 months), mid-stage (0–6 months) and late-stage (0–6 months+). Eight studies investigated through to the late phase [32, 35, 39, 40, 44, 45, 48, 49], nine mid-stage [30, 31, 33, 36, 41–43, 46, 47] and two early-stage [37, 38]. Only five studies had a follow-up assessment period longer than the intervention period [31, 34, 37, 41, 47].

Thirteen of the included 20 studies showed no significant difference between low, moderate or high frequency supervised rehabilitation regardless of the intervention period. The non-significant studies were all seven RCTs [31, 37, 38, 40, 45, 46, 48], five retrospective studies [34, 35, 41, 47, 49] and one prospective study [36]. Seven studies, all published in the last 4 years, showed an association between improved outcome and moderate or high-frequency supervised rehabilitation. Specifically, prospectively designed studies found associations between proprioception recovery [42], functional knee movement [42], higher return to preinjury level of sports [44] and better quality of life [44] in a highly supervised group than in a low supervision home-based group.

Studies utilising a retrospective methodology found an association between higher rehabilitation utilisation and significantly higher patient reported outcomes (Knee Outcomes Survey–Activities of Daily Living (KOS-ADL) scale [32, 43], Knee Injury and Osteoarthritis Outcome Score subscales [39], patient satisfaction [47] and numerical pain rating scale [43]), greater return to pre-operative activities [39, 47] and improved Lysholm knee score [39, 47]. Finally, in a cross-sectional study, Dempsey et al. (2019) found a weak positive correlation with isokinetic knee extension torque and level of supervision [30], while Darain et al. (2015) demonstrated a successful return to sport at 6 months with a high frequency of supervised rehabilitation in a case report [33].

#### Summary

Despite significant heterogeneity between the included studies and overall poor quality of research, it is reasonable to conclude that a moderately or minimally supervised rehabilitation program is at least as effective as a fully supervised high-frequency rehabilitation program. Recent publications, however, are showing an association between higher rehabilitation utilisation improving outcomes. It remains to be seen whether there is an optimal frequency of supervised rehabilitation visits and if this varies between stages of rehabilitation. From the current research, it is unclear whether participants met an acceptable level of function for return to sport and minimisation of reinjury.

### Supervised Rehabilitation Duration

#### Original Studies

Of the eight included original studies regarding supervision duration, seven studies are retrospective and one prospective in design (Table 4). The average age was 27.9 (26.2–29.7). Seven studies utilised a hamstring graft, while one study used both BPTB and hamstring graft. Participant activity level was reported in all but one study but was largely poorly defined. All studies compared a group of patients who completed a shorter duration of supervised rehabilitation (less than 3 or 6 months) to a group of patients who completed six or more months of rehabilitation, including structured agility, gym exercises, landing and on-field rehabilitation in line with current evidence-based recommendations

**Table 4:** Summary of included original studies investigating ‘supervised rehabilitation duration’

Author (year)	Design	Population					Investigation	OM assessment (months)	OMs
		*n*	Age (mean)	Sex	Graft	Activity level			
Ebert et al. (2019) [50]	Retrospective	111	27.3	M 73 F 38	HS	Noyes level 1 or 2 sports participation	Level of post-operative rehabilitation completed (7 point scale)	12.5 (at RTS)	NSARS, single hop for distance, triple hop for distance, 6 m timed hop, triple crossover hop for distance, isokinetic Fl and ext (90deg/sec)
Edwards et al. (2017) [51]	Retrospective	113	27	M 75 F 38	HS	Noyes level 1 or 2 sports participation	Level of post-operative rehabilitation completed (7 point scale)	10-14	IKDC subjective knee evaluation, NSARS, single hop for distance, triple hop for distance, 6m timed hop, triple crossover hop for distance, isokinetic Fl and ext (90deg/sec), pass/fail of the test battery
Krolikowska et al. (2018) [52]	Retrospective	38	29.7	M 38	HS	Sports participation	Supervised rehabilitation < 6 months and > 6 months	Average of 2 years	IKDC knee evaluation form, DL and SL vertical hop analysis, Lachman’s, pivot shift
Krolikowska et al. (2018) [53]	Prospective	35	27.3	M 35	HS	Not stated	Supervised rehabilitation < 6 months and > 6 months	7 (end of stage 4)	IKDC knee evaluation form, DL and SL vertical hop analysis
Krolikowska et al. (2019) [54]	Retrospective	143	29.3	M 143	HS	Sports participation (but not high level)	Supervised rehabilitation < 6 months and > 6 months	7 (end of stage 4)	Isokinetic Fl (180deg/sec)
Krolikowska et al. (2018) [55]	Retrospective	30	26.1	M 30	HS	Sports participation	Supervised rehabilitation < 3 months and > 6 months	8	IKDC knee examination Form, VAS pain, agility test (speed and time)
Krolikowska et al. (2018) [56]	Retrospective	30	26.2	M 30	HS	Sports participation (but not high level)	Supervised rehabilitation < 3 months and > 6 months	8	Isokinetic Fl and ext (180deg/sec and 60deg/sec)
Rosso et al. (2018) [57]	Retrospective	174	29.5	M 141 F 35	HS/BPTB	Sports participation	Supervised rehabilitation < 3 months and > 3 months	3.75 years	IKDC knee evaluation form, pivot shift, Lachman’s, anterior crackling, single hop for distance, Lysholm knee score, IKDC subjective knee evaluation, return to sport status, SPORTS score, ACL-RSI, re-rupture

*ACL* anterior cruciate ligament, *ACL*-*RSI* anterior cruciate ligament—return to sport after injury, *BPTB* bone patella tendon bone, *deg*/*sec* degrees per second, *DL* double leg, *ext* extension, *F female*, *Fl* flexion, *HS* hamstring, *IKDC* International knee documentation committee, *LSI* limb symmetry index, *m* metre, *M* male, *NSARS* Noyes sports activity rating scale, *SL* single leg, *SPORTS* subjective patient outcome for return to sports

Supervised rehabilitation longer than 6 months was associated with improved outcomes at all assessment time points. Specifically, associations were found between longer supervised rehabilitation and functional symmetry [50, 51], a greater likelihood of meeting return to sport criteria and RTS at 12 months [50, 51], double leg vertical hop landing symmetry [52, 53], knee flexor rate of torque development and symmetry [54], speed and agility [55], knee extensor muscles torque parameters and LSI values [56] and better subjective outcomes [57]. Delaying the start of rehabilitation longer than one month after reconstruction was negatively associated with objective outcomes [57]. However, the duration of supervised rehabilitation was not associated with one leg vertical hop symmetry [53], knee joint stability, thigh and knee joint circumferences, active range of motion or everyday pain [55].

#### Summary

It is reasonable to conclude that a longer duration of supervised rehabilitation of at least 6 months, which includes structured agility, gym exercises, landing and on-field rehabilitation, is associated with more favourable outcomes after ACL reconstruction. It is likely that 9 or 12 months of structured supervised rehabilitation would offer further benefits. High-quality prospective randomised trials in this area are required.

### Adherence and outcome

#### Reviews

All three reviews evaluated adherence to clinic and home-based rehabilitation against functional and subjective outcomes (Table 5). There is an overall lack of evidence in the area of adherence and its effect on rehabilitation. Two reviews demonstrated a positive relationship between greater adherence to rehabilitation and improved outcomes [58, 60], while one review was inconclusive [59]. The methodological quality of included studies is uncertain as it has yet to be evaluated appropriately.

**Table 5:** Summary of included reviews investigating 'adherence and outcome’

Author (year)	Review type	Dates	Methodological quality	No. Included original studies	Conclusion
Christino et al. (2015) [58]	Non-systematic	Not specified	Not assessed	N/A	Positive correlation
Mendonza et al. (2007) [59]	Systematic	Until 2006	Not assessed	3	Inconclusive
te Wierike et al. (2013) [60]	Systematic	2001–2011	Good	1	Positive correlation

Methodological quality refers to the outcome or presence of a quality appraisal undertaken by the review not the authors of this study. The conclusion stated is that of the included review in reference to the correlation between adherence and rehabilitation outcome. The number of original studies is only those included in each review in the evaluation of adherence and outcome

#### Original studies

Of the four included original studies regarding adherence, three studies of prospective design compared measures of adherence to clinic and home-based rehabilitation against functional and subjective outcomes over the first 6 weeks, 8 weeks and 6 months of rehabilitation (Table 6). The average age was 28.4 (26.9–29.4). One study used both BPTB and hamstring graft; the other two studies did not state. Participant activity level was stated in two studies. Outcomes were assessed at six months and 9–12 months.

**Table 6:** Summary of included original studies investigating ‘adherence and outcome’

Author (year)	Design	Population					Investigation	Adherence measure	OM assessment (months)	OMs
		n	Age (mean)	Sex	Graft	Activity level				
Brewer et al. (2004) [63]	Prospective	108	29.4	M 72 F 30	Not stated	Not stated	Adherence to clinic and home-based rehabilitation in the first 6 weeks	Adherence to appointments (% attended of scheduled) Adherence to home-exercise prescription (self-report diary and hidden tape play counter) Adherence during appointments (SIRAS)	6	Single leg hop for distance, Lachmans, KOOS-SAS
Brewer et al. (2000) [62]	Prospective	95	26.9	M 67 F 28	Not stated	Competitive (52%) Recreational (43%) Non-athletes (3%)	Adherence to clinic and home-based rehabilitation In the first 6 months	Adherence to appointments (% attended of scheduled) Adherence to home-exercise prescription (0–10 self-report adherence) Adherence during appointments (SIRAS)	6	Lysholm knee score, KT-1000, single hop for distance
Levinger et al. (2017) [61]	Pilot RCT	17	32.2	M 9 F 8	Not stated	Not stated	Adherence to a web informational support service	Adherence to recommended website usage (% out of 22)	3	KOOS, K-SES, FABQ, TSK-SF, IPAQ, qualitative interview
Pizzari et al. (2005) [64]	Prospective	68	28.8	M 48 F 26	HS/BPTB	Competitive (63%)	Adherence to clinic and home-based rehabilitation in the first 8 weeks	Adherence to appointments (% attended of scheduled) Adherence during appointments (SIRAS) Adherence to home-exercise prescription (self-report diary, % exercises completed)	9-12	IKDC knee evaluation form, IKDC subjection knee evaluation, CKRS, KOOS, 6m timed hop, triple crossover hop for distance

*CKRS* Cincinnati knee rating system, *F* female, *FABQ* Fear-avoidance beliefs questionnaire, *IKDC* International knee documentation committee, *IPAQ* International physical activity questionnaires, *K*-*SES* knee self-efficacy scale, *KOOS* knee injury and osteoarthritis outcome score, *KOOS*-*SAS* knee injury and osteoarthritis outcome score-sports activity scale, *M* male, *m* metre, *OMs* outcome measures, *RCT* randomised controlled trial, *SIRAS* sports injury rehabilitation adherence scale, *TSK*-*SF* Tampa scale for kinesiophobia- short form

It is inconclusive whether adherence has a positive effect on outcome. Significant correlations have been demonstrated between greater adherence to clinic-based rehabilitation and improved Knee Outcomes Survey–Sports Activities Scale scores [63] and one leg hop [62]. On the contrary, no significant correlation was found between any OMs and adherence measures in one study [64] and Brewer et al. (2004) found greater adherence to clinic-based rehabilitation was associated with high Lachman’s grade [63]. Adherence to home-based rehabilitation negatively predicted Cincinnati Knee Rating System- Sport scores [64] and was a negative correlate to all OMs for participants > 30 years, but a positive correlation if < 30 years [64]. There was also no difference in any outcome measure with adherence to a web informational support system, despite the intervention group reporting being more committed to rehabilitation [61].

#### Summary

When considered with the results of the included reviews and the conflicting results of the few original studies investigating adherence to clinic and home-based rehabilitation against outcomes, an overall conclusion cannot be made on the effects of adherence to rehabilitation and outcome.

## Category (B) Rehabilitation Barriers and Facilitators

### Psychological

#### Reviews

The 10 reviews reported on 19 different psychological variables (Table 7). All of the variables could either act as a barrier or a facilitator to rehabilitation depending on the individual patient. For example, high self-motivation is considered to facilitate rehabilitation, while low self-motivation may act as a significant barrier to rehabilitation.

**Table 7:** Summary of included reviews investigating ‘psychological’

Author (year)	Design	Dates	Methodological quality	Included	Key concepts
Anderson et al. (2016) [13]	Systematic	2004–2014	Not assessed	2	Self-confidence, optimism, self-motivation, stress, social support, athletic identity
Ardern et al. (2016) [66]	Narrative	Not specified	Not assessed	N/A	Cognitive appraisal, previous experiences, attitudes, self-efficacy, fear of reinjury
Burland et al. (2019) [67]	Narrative	Not specified	Not assessed	N/A	Self-efficacy
Christiano et al. (2015) [58]	Narrative	Not specified	Not assessed	3	Self-esteem, post-traumatic stress, pain intolerance, mood disturbance, goal setting, positive self-talk, fear of reinjury, self-efficacy, self-motivation, athletic identity
Everhart et al. (2015) [65]	Systematic	1975–2012	63/90 modified Coleman score	8	Goal setting, positive self-talk, self-motivation, self-efficacy, optimism, self-confidence, stress, social support, athletic identity
Flanagan et al. (2015) [68]	Narrative	Not specified	Not assessed	N/A	Self-efficacy, self-motivation, athletic identity, social support, fear of reinjury, confidence, optimism/pessimism, goal setting, positive self-talk
Mendonza et al. (2007) [59]	Systematic	Until 2006	Not assessed	7	Self-motivation, social support, athletic identity, goal setting, positive self-talk, situational stability, stability, personal control
Sims and Mulcahey (2018) [69]	Narrative	Not specified	Not assessed	N/A	Self-confidence, optimism, self-motivation, locus of control, athletic identity, sex difference in psychology
Spetch and Kolt (2001) [21]	Narrative	Not specified	Not assessed	N/A	Stress, self-motivation
te Wierike et al. (2013) [60]	Systematic	2001–2011	Good	2	Self-efficacy, age and sex effect on psychology, low confidence/self-esteem, social support, goal setting, positive self-talk, avoidance coping

Methodological quality refers to the outcome or presence of the quality appraisal undertaken by the review not the authors of this study. The key concepts list all of the psychological concepts addressed by each review in relation to psychological variables and adherence to rehabilitation

The most to least commonly reported psychological variables were self-motivation [13, 21, 58, 59, 65, 68, 69], athletic identity [13, 58, 59, 65, 68, 69], self-efficacy [58, 60, 65–68], self-confidence [13, 60, 65, 68, 69], positive self-talk [58–60, 65, 68], social support [13, 59, 60, 65, 68], optimism [13, 65, 68, 69], goal setting [59, 60, 65, 68], stress [13, 21, 58, 65], fear of reinjury [58, 66, 68], locus of control [59, 69], age and sex differences in psychology [60, 69], self-esteem [58], pain tolerance [58], mood disturbance [58], situational stability [59], cognitive appraisal [66] and coping strategies [60], previous experiences [66] and attitudes [66]. As most of the reviews are narrative in nature and with a low overall evidence base (8 original studies), most of the conclusions within each review are theoretical in nature and were drawn from a wider evidence base from other injuries and disciplines. This provides scope for further research in this area in the ACL reconstruction population.

#### Original Studies

Of the eight included original studies regarding psychological factors, six studies were prospective in design, one retrospective and one case series (Table 8). The average age across the studies was 28.3 (25.2–32). All studies had male and female participants, and all but one study stated the participant activity level.

**Table 8:** Summary of included original studies investigating ‘psychological’

Author (year)	Design	Population				Concepts investigated	OMs	Adherence measures
		*n*	Age (mean)	Sex	Activity level			
Brewer et al. (2003) [73]	Prospective	61	26	M 40 F 21	Competitive (57%) Recreational (41%) Non-athletes (3%)	Self-motivation Athletic Identity Social support	SMI, SSI, AIMS, BSI	Rehabilitation attendance index (attended/scheduled), SIRAS, self-reported home rehabilitation adherence (0–10 scale)
Brewer et al. (2013) [75]	Prospective	91	29.7	M 58 F 33	Competitive (57%) Recreational (41%) Non-athletes (3%)	Stress and mood Athletic identity Neuroticism Pessimism	AIMS, NEO-FFI, PESS, LOT-R, POMS-B, Lysholm knee score, NPRS, daily stress 0-5 scale	Self-reported home rehabilitation adherence (0–10 scale)
Brewer et al. (2000) [62]	Prospective	95	26.9	M 67 F 28	Competitive (52%) Recreational (43%) Non-athletes (3%)	Self-motivation Athletic Identity Social support	SMI, SSI, AIMS, BSI, KT1000, single leg hop for distance	Rehabilitation attendance index (attended/scheduled), SIRASSelf-reported rehabilitation adherence diary (0–10 scale)
Chan et al. (2003) [74]	Retrospective	95	25.2	M 94 F 21	100% sport participation	Autonomy Self-motivation	HCCQ, TSRQ	Combined items from the SIRAS and Modified Patient Self-Report Scales of Their Home-based Rehabilitation Adherence
Hilliard et al. (2004) [76]	Prospective	108	29.4	M 72 F 36	Not stated	Personality traits	NEO-FF	SIRAS, Rehabilitation attendance index (attended/scheduled)
Niven et al. (2012) [70]	Prospective	87	29	M 65 F 28	International (10%) National (12%) District (14%) Club (45%) Recreational (18%)	Theory of planned behaviour (self-efficacy, intention)	Attitudes towards ACL Rehabilitation Questionnaire	Self-reported rehabilitation adherence (0–7 scale)
Rock and Jones (2002) [71]	Case series	3	32	M 1 F 2	Competitive athletes	Social support Self-motivation Counselling	SSBS, emotion scores in ERAIQ, NPRS, perceived rehabilitation completion (0-100 scale)	SIRAS
Scherzer et al. (2001) [72]	Prospective	54	28	M 37 F 17	Competitive (52%) Recreational (46%) Non-athletes (2%)	Goal setting Imagery Positive self-talk	Sports Injury Survey subscales (goal setting, healing imagery, positive self-talk)	Rehabilitation attendance index (attended/scheduled) Self-reported rehabilitation adherence (0-10 scale)

*AIMS* athlete identity measurement scale, *BSI* brief symptom inventory, *ERAIQ* emotional response of athletes to injury questionnaire, *HCCQ* health care climate questionnaire, *LOT*-*R* the life orientation test-revised, *NEO*-*FF* neuroticism five-factor inventory, *NPRS* numerical pain rating scale, *PESS* pessimism, *POMS*-*B* negative mood–profile of mood states-B, *SIRAS* sports injury rehabilitation adherence scale, *SMI* self-motivation inventory, *SSBS* social support behaviours survey, *SSI* social support inventory, *TSRQ* treatment self-regulation questionnaire

Twelve psychological concepts were investigated, utilising a variety of concept specific outcome measures to determine their correlation with adherence to clinic and home-based rehabilitation (Table 8). These were self-motivation [62, 71, 73, 74], athletic identity [62, 73, 75], social support [62, 71, 73], stress and mood disturbance [75], neuroticism [75], pessimism [75], autonomy [74], personality traits [76], the theory of planned behaviour [70], counselling utility [71] and goal setting, imagery and positive self-talk [72]. Their correlation with rehabilitation adherence is detailed below and varies depending on the concept, age of participants or the setting of rehabilitation (clinic or home based).

Self-motivation was associated with home exercise completion [62, 71, 74]; this was true for older participants only in one study [73]. A high athletic identity in younger patients was associated with home exercise completion [73]. However, in two studies, athletic identity was not correlated with adherence to clinic or home-based exercise [62, 75]; except on days with high-stress, participants with high athletic identity completed more exercises [75].

Social support was not found to be significantly related to home exercise completion [62], except in older participants [73]. High stress and mood disturbance were negatively associated with home exercise completion. Neuroticism was not related to adherence, and participants with low pessimism were able to complete more prescribed exercises on days where they had more pain [75]. Goal setting and positive self-talk were significant positive correlates to home exercise adherence [72]. These were not related to clinic attendance or cryotherapy completion [72]. Autonomy had a positive relationship with rehabilitation adherence [74], and the Big 5 personality traits of agreeableness and conscientiousness were significantly correlated with adherence measures [74]. The theory of planned behaviour [70], imagery [72] and counselling sessions [71] did not correlate with rehabilitation adherence or participation.

#### Summary

There are a variety of psychological variables which may affect a person’s adherence to rehabilitation; however, we did not investigate whether interventions to address these factors would lead to an increase in adherence. Further research aimed at addressing these factors and the effect that they have on rehabilitation adherence and subsequent patient outcomes is warranted.

### Patient Perceptions

Table 9 details the thematic synthesis of patient-perceived barriers to and facilitators of rehabilitation. Fifteen original studies were included in the analysis. Eight studies used a qualitative methodology [15, 20, 80–85], four mixed methods [30, 77–79], one pilot RCT [61], one case series [71] and one prospective cohort study [86]. Fifty-five raw themes were categorised into three overall themes (environmental, personal and treatment-related) and nine sub-themes as detailed in Table 10.

**Table 9:** Frequency of mention count for each theme identified in the synthesis of included original studies investigating ‘patient perceptions’

Factor	Barrier	Facilitator	Total
Environment	**11**	**8**	**19**
Social	**6**	**8**	**14**
Interaction with family and friends	3	6	9
Interaction with team and coaches	3	2	5
Societal	5	0	5
Access to facilities and equipment	4		4
Access to skilled providers	1		1
Personal	**28**	**23**	**51**
Psychological	**16**	**15**	**31**
Fear	7		7
Self-motivation (low/high)	4	5	9
Met or unmet expectations	2	2	4
Restlessness and impatience	1		1
Hopelessness/Belief	1	1	2
Previous experience (bad/good)	1	1	2
Progress changeability		1	1
Acceptance		1	1
Positive attitude		2	2
Feeling appreciated		1	1
Luck		1	1
Physiological	**6**	**2**	**8**
Pain, weakness and illness	4		4
Significant injury	1		1
Second injury	1		1
Maintain health and fitness		2	2
Behavioural	**6**	**6**	**12**
Organisation/lack of time (poor/good)	5	1	6
Goal setting	1	2	3
Persistence		2	2
Distraction (new activities)		1	1
Treatment-related	**36**	**46**	**82**
Delivery of care	**18**	**13**	**31**
Length and commitment of rehabilitation	4		4
Non-sport specific exercise	2		2
Restrictions in activities	2		2
Enjoyment	2	2	4
Patient control (low/high)	2	1	3
Insurance	2	1	3
Assessment of progress	1	3	4
Speed of progression of exercises (slow/fast)	1	1	2
Early therapist discharge	1		1
Cost	1		1
Individualised program		3	3
Comfort and convenience		1	1
Cryotherapy		1	1
Provider factors	**12**	**13**	**25**
Therapeutic relationship	4	7	11
Physiotherapist as a guide and coordinator	2	2	4
Coordination between providers	3	2	5
Information availability	3	2	5
Digital health	**3**	**8**	**11**
Poor accessibility	1		1
Uncertainty of technique and safety	1		1
Familiarity with digital devices	1	1	2
Blended care model		2	2
Informational and instructive		2	2
Reminder for exercise completion		2	2
Viewed as the future		1	1
Group rehabilitation	**3**	**12**	**15**
Interpersonal comparison	2	4	6
Social interaction	1	1	2
Informational support		2	2
Fun and enjoyable		1	1
Motivation and support		1	1
Innovative		1	1
Obligation		1	1
Adequate monitoring and adaptability		1	1

Bold text highlights each key theme and sub-theme

Each theme was tallied on the number of times it was mentioned across the literature. A theme was only tallied once per article. The tally does not imply the weight of the barrier or facilitator on the subjects but only how often the factor has emerged in the research. Under the personal category, any theme relating to the mental and emotional state of a person, affecting, or arising in the mind was classified as psychological. However, any theme involving, or relating to, exhibiting a behaviour was categorised as behavioural. A factor could be both a facilitator and barrier. For example, interaction with family and friends may be a barrier if it involved sympathy, caution and worry from family and friends, but a facilitator if it involved support, motivation and encouragement.

Treatment-related factors were mentioned 82 times across three sub-themes (delivery of care, digital health and group rehabilitation) consisting of 32 raw themes; 36 mentions as a barrier to and 46 mentions as a facilitator of rehabilitation. Personal factors were mentioned 51 times across three sub-themes (psychological, physiological and behavioural), consisting of 19 raw themes; 28 mentions as a barrier and 23 mentions as a facilitator. Environmental factors were mentioned 19 times across two sub-themes (social and societal), consisting of four raw themes; 11 mentions as a barrier and eight mentions as a facilitator.

The most common raw themes arising in the literature as either a barrier or facilitator of rehabilitation were therapeutic relationship (*n* = 11), interaction with family and friends (*n* = 9), self-motivation (*n* = 9), fear of reinjury or return to sport (*n* = 7), organisation/lack of time (*n* = 6), interpersonal comparison (*n* = 6), interaction with team and coaches (*n* = 5), access to facilities and equipment (*n* = 4), expectations (*n* = 4), pain, weakness or illness (*n* = 4), length and commitment of rehabilitation (*n* = 4) and enjoyment (*n* = 4).

#### Summary

These results signify the key role the treating health practitioner plays in setting an appropriate rehabilitation environment to reduce treatment-related barriers to and enhance facilitators of rehabilitation but also support the athlete with a strong therapeutic relationship which fosters motivation and enjoyment. Specific personal factors related to the individual may be able to be addressed through therapeutic exercises (e.g. fear of reinjury) or may require tailored interventions or alternative professionals to facilitate rehabilitation. Social and societal factors also play a key role but are harder to influence by the practitioner.

### Other factors

The three included original studies investigated associations between clinician experience and qualification [87], graft choice and meniscal injury [30] and participant sport [70] on rehabilitation adherence (Table 10).

**Table 10:** Summary of included original studies investigating ‘other factors’

Author (year)	Design	Population				Concepts investigated	OMs	Comparators
		*n*	Age (mean)	Sex	Activity level			
Dempsey et al. (2019) [30]	Mixed methods	60	29.7	M 31 F 29	Competitive (57%) Recreational (41%) Non-athletes (3%)	Graft type Meniscus injury	Sessions attended	BPTB or HS graft Meniscus injury
Greenberg et al. (2018) [87]	Cross-sectional	1074	N/A	N/A	N/A	Physiotherapist practice patterns	N/A	N/A
Niven et al. (2012) [70]	Prospective	87	29	M 65 F 28	Not stated	Level and type of sport	Attitudes towards ACL Rehabilitation Questionnaire	Self-reported rehabilitation adherence (0–7 scale)

*M* male, *BPTB* bone patella tendon bone, *F* female, *HS* hamstring, *OCS* orthopaedic certified specialist, *SCS* sport certified specialist

To determine physiotherapist practice patterns, Greenberg et al. (2018) surveyed 1074 physiotherapists from the USA. They found clinicians with less clinical experience, higher volumes of patients post ACL reconstruction and an orthopaedic clinical specialist or sports clinical specialist certification deliver a longer overall duration of clinical care more in line with clinical recommendations [87].

In terms of graft choice and meniscus injury, Dempsey et al. (2019) found that competitive and recreational athletes who received a BPTB graft completed more days of rehabilitation per week and had more total visits compared with patients who received an HT graft; however, meniscal procedures did not correlate with rehabilitation quantity [30].

In a prospective study, Niven et al. (2012) found variation in the adherence levels across different sports, indicating that Gaelic football, hockey, rugby and soccer players consistently adhered well, whereas motocross participants were poor adherers [70]. The level of sport had a positive relationship, indicating that a lower level of participation was associated with higher adherence levels [70].

#### Summary

Newly graduated and specialty trained therapists may be more cognisant of current evidence and delivery care more in line with current recommendations. Although patients with BPTB graft attended more often, it is unclear what this may be due to and the implications for rehabilitation adherence. Finally, it is unclear as to the reasons why different sports have different levels of adherence.

## Discussion

Participation in ACL rehabilitation is considered critical to facilitate return to sport [14, 51, 88]. In this scoping review, 71 articles relating to adherence and participation in ACL rehabilitation published between 1997 and 2019 were identified. A key finding of this review was that a longer duration of supervised evidence-based rehabilitation is correlated with more favourable outcomes post ACL reconstruction; however, the optimal frequency of rehabilitation supervision and the level of adherence required to a rehabilitation program is yet to be determined. It is reasonable to conclude that from current evidence, a minimally or moderately supervised rehabilitation program is at least as effective as a fully supervised high-frequency rehabilitation program.

Furthermore, many factors were associated with a patient’s ability to adhere to and participate in rehabilitation. Psychological factors of self-motivation, athletic identity, stress and mood disturbance, goal setting, positive self-talk and the personality traits of optimism, agreeableness and conscientiousness were associated with rehabilitation adherence. Numerous patient-perceived barriers to and facilitators of rehabilitation were identified. The most common were the therapeutic relationship, interaction with family and friends, self-motivation, fear of reinjury and organisation/lack of time.

For the researcher and clinician, the results of our scoping review highlight the need to develop appropriate rehabilitation protocols that not only develop the physical capabilities of patients but also take into account patients’ circumstances and psychology, which may pose barriers to achieving a successful outcome. Aspects of rehabilitation may need to be varied depending on the individual presenting.

### How Much Supervision Is Required, and For How Long?

It would be premature to conclude that reducing rehabilitation supervision during ACL reconstruction is required. Current practice patterns in Australia reflect a decreasing frequency of supervised rehabilitation from once or twice per week in the early phases, to less frequent visitation with a focus on independent exercise with periodic review as rehabilitation progresses [89]. The evidence in this review is inconclusive as to whether this is the most appropriate way to manage patients.

Based on our results, the duration of supervised rehabilitation may be more important than frequency. Supervised rehabilitation should begin shortly after surgery [57], continue for greater than 6 months (ideally 9–12 months) and include a tailored gym program, landing, agility, on-field rehabilitation and a structured return to sport. It appears that two patients performing the same rehabilitation program can achieve the same outcome regardless of supervision or adherence level; however, it remains to be seen whether patients have the knowledge and skills to complete rehabilitation at the appropriate intensity to achieve return to sport criteria without appropriate supervision [55].

Recent original studies have demonstrated that even with well-controlled and implemented rehabilitation, most athletes fail to meet discharge criteria [90]. Furthermore, in the community, only 30% of patients complete any form of rehabilitation beyond 6 months [91] and only 5% of people complete evidence-based rehabilitation. Edwards et al. (2017) demonstrated only 21% of patients who had completed rehabilitation and 5% of patients who had not completed rehabilitation passed a RTS test battery before RTS [46]. Therefore, even if patients do complete rehabilitation, the end phase of rehabilitation is typically not extensive or specific enough, failing to expose patients to specific training loads and training characteristics necessary before they return to unrestricted sport [90]. Due to the knowledge and skills required to execute late-stage rehabilitation to a sufficient standard and intensity, a higher level of supervision may be needed in the later phases to meet return to sport criteria and reduce the risk of reinjury [50, 51, 55].

All original studies which showed a positive relationship between supervised rehabilitation frequency and outcomes were published in the last 4 years. This fact may suggest that modern rehabilitation programs may require more guidance from a clinician. Age may also play a role in the frequency of supervision required. Younger patients, particularly under 18, may require a higher frequency to achieve successful outcomes by providing extra guidance on exercises, goals and motivation to adhere to post-operative rehabilitation [43, 46, 64].

Clinician knowledge may also play a role in achieving a successful outcome. Clinicians familiar with current best practice who service a higher volume of patients who have undergone ACL reconstruction are more likely to provide evidence-based care, while less familiar clinicians may be at risk of prematurely discharging patients before meeting established RTS criteria [87]. This may be due to a lack of confidence, skills or resources in the performance of late stage rehabilitation and return to sport criteria. Clinicians, therefore, need to be aware of their own limitations and potentially refer to other health professionals.

A final point to consider is that the increasing demand for cost-effective health care interventions is leading to the development of more unsupervised rehabilitation protocols [25]. Rehabilitation needs to be both effective and economical. There are substantial financial advantages of more patient-directed rehabilitation in reducing costs for the appointment, travel time, inconvenience, time off work and comfort [25, 34, 35]. This presents a tough challenge for clinicians to ensure that patients have access to appropriate rehabilitation to achieve functional and sporting goals, but not increase the undue financial burden upon the patient, health care system and industry [37]. No articles in this review included a cost-benefit analysis, which would aid in the development of a more robust research base and allow us to gain further insight into how to minimise costs and maximise outcome.

When designing future research to examine the adherence and outcome relationship, it is critical that researchers consider the definition of adherence, parameter, adherence measure and the value for acceptable adherence [91]. The studies detailed in this review [61–64] used a variety of measures including self-report diary, sessions attended, adherence within session (SIRAS) and hidden tape player counters. Multiple systematic reviews have highlighted a lack of a single valid and reliable measurement tool of adherence means that the relative effectiveness of interventions is difficult to compare across studies [92–94]. In a recent systematic review by Bailey et al. (2018) [91], the authors concluded there is a lack of sufficient consistency in adherence parameters, measures and values to inform a definition of adherence to therapeutic exercise or the required content of a suitable measure. The definition by Frost et al. (2017) (‘the extent to which individuals undertake prescribed behaviour accurately and at the agreed frequency, intensity and duration’ [95]) is provided as a starting point to develop an appropriate adherence measure as it includes the measures of frequency, duration, intensity and accuracy [91].

### Can We Improve Rehabilitation Adherence and Participation?

Despite our improved understanding of what components need to be included within an evidence-based ACL rehabilitation [14], little consideration is given to why patients cease rehabilitation and the barriers which patients face in their rehabilitation journey [30].

Psychological factors, particularly fear of reinjury, are the most significant contributor to not returning to sport [96]. The results of this review support the notion that psychological variables contribute to patients ceasing or failing to adhere to rehabilitation. Self-efficacy was consistently reported as a significant mediator of successful surgery and rehabilitation [61]. Strategies to enhance patients’ self-efficacy have the potential to improve related barriers to participation, such as self-confidence, locus of control, autonomy support and stress and mood disturbance [97]. Likewise, the enhancement of patient self-motivation improves the chance they will persist with rehabilitation [98]. Patients can draw extrinsic motivation from the physiotherapist and rehabilitation program (e.g. progressing exercises, reassessing progress, goal setting, social and informational support) and therefore, increase their likelihood to participate in and progress through an appropriate duration of rehabilitation [58, 59]. Put together with the appropriate progression of exercises to expose patients to psychologically challenging but safe situations, fear of reinjury could also be reduced, increasing the likelihood of a return to sport and reducing reinjury [60]. Clinicians also need to be aware that some patients may require referral to an appropriate health care professional to receive specialised psychological care.

Especially due to the long rehabilitation process, by structuring or delivering rehabilitation in a manner that supports a positive psychological state (managing mood disturbance, enhancing and maintaining athletic identity and utilisation of goal setting and self-talk), many barriers to rehabilitation and return to sport can be overcome [60, 81, 85]. Further research into the utility of psychological intervention in ACL rehabilitation is needed [99].

### How Can the Clinician Help the Patient?

**Fig. 3:**
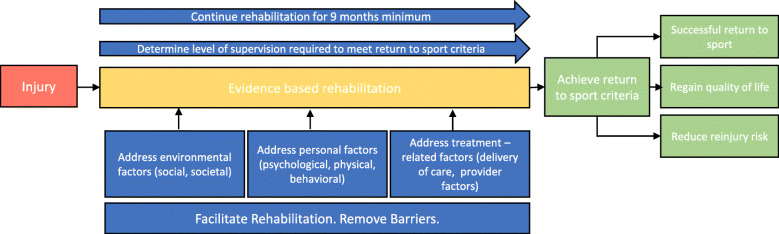
Thematic model depicting individual considerations required when planning ACL rehabilitation

Our results show a large number of patient-perceived barriers to and facilitators of rehabilitation. Many of the factors likely interact with each other, and by putting in place practices that either enhance facilitators or remove barriers, outcomes could be improved (Fig. 3).

As exemplified in our results, the physiotherapist plays a significant role in driving recovery by offering motivation, support, guidance, and encouragement while also providing informational support. It is also the physiotherapist’s responsibility to set realistic expectations and deliver a fun, progressive, sport-specific program for the individual with regular goal setting and reassessment. The physiotherapist can also assist in overcoming the physiological barriers to rehabilitation, such as pain and reduced health and fitness due to injury.

Group-based rehabilitation has the potential to overcome many of the barriers to and enhance facilitators of rehabilitation. Studies investigating group-based therapy demonstrated positive characteristics, including an enjoyable, cost-effective, social, supportive and motivating environment [80, 85]. Particularly with advancements in technology, the support of digital health technology is becoming increasingly valuable. By supporting face to face interactions, it provides a useful adjunct to improve exercise adherence, increase engagement, enhance the therapeutic relationship and provide informational support to what is required at each stage, assisting in setting realistic expectations [81].

Environmental factors are harder to control as they are often out of the control of the treating physiotherapist. Physiotherapists working within team environments can assist by providing coaches and teammates with the appropriate information to facilitate inclusion and interaction with the main training group. If the physiotherapist has interaction with family members and friends, positive supportive behaviours can also be reinforced. Geographic constraints that prevent access to appropriate facilities and providers pose a particular challenge. Digital health may be an area of future research to address this domain.

### Strengths and Limitations

This review is the first to address the effects of rehabilitation adherence and participation on ACL rehabilitation outcome. We were then able to provide the reader with potential influencing factors which create barriers to or facilitate rehabilitation. The review was also conducted according to recognised standards for scoping review following the development and publication of an a priori protocol.

The methodological quality of the articles was not assessed as per guidelines for conducting scoping reviews [16, 17]. Many studies were deemed as methodologically poor in quality, suggesting that more work is needed in developing good quality research in this area. There was no date limit on the search or inclusion. Included articles may not reflect contemporary practice due to changes in practice patterns through time.

When assessing the evidence for the frequency of supervised rehabilitation, it was not determined whether any cohort of patients achieved a successful outcome from their rehabilitation. Lynch et al. (2013) detailed the criteria for defining a successful outcome after ACL reconstruction. These are the absence of giving way, patient return to sport status, the absence of knee joint effusion, quadriceps muscle strength symmetry and meeting patient-reported outcome benchmarks [100]. Due to the outcome measure heterogeneity, it was not possible to evaluate whether a successful rehabilitation outcome was achieved. The level of compliance of patients in the included original studies within the frequency analysis to the home-based rehabilitation prescribed was also unknown [23].

Only articles published in English were available for inclusion, introducing a publication bias. Only one author screened, selected and extracted the data from the studies, potentially missing articles or introducing bias to data presented. The articles were categorised and analysed based on the author determined constructs. The categorisation may have been different for different authors.

Considerable heterogeneity between studies in outcome measures used, rehabilitation timeframes and programs reduced the ability to compare results directly. Most studies reported on participants over the age of 25, reducing the ability to draw conclusions for patients in a younger age group who typically have higher return to sport goals. Poor reporting of activity level and sport of the included participants leads to uncertainty in identifying factors relevant to specific athletes, sports or activity levels. Studies were from a variety of countries, introducing biases into the results due to different standards of care and access to health services. However, the review provides a comprehensive analysis of the current state of knowledge and areas where further work is needed to facilitate better rehabilitation practices.

## Conclusion

This scoping review highlighted a broad spectrum of factors the clinician should consider when facilitating a patient’s rehabilitation after ACL reconstruction. Growing evidence suggests a longer duration of supervised rehabilitation involving agility, landing and gym exercises, and a supervised return to activity or sport is required to achieve functional and return to sport goals. The lack of conclusive evidence to support a specific supervised rehabilitation frequency fails to provide appropriate guidance to treating physiotherapists to deliver more optimal care.

Identification of the barriers to and facilitators of adherence and participation in ACL rehabilitation provides an opportunity for further research to be conducted to address personal, environmental, and treatment-related factors. Taking these factors into account increases the likelihood of patients complying with current best evidence rehabilitation to improve outcomes such as return to sport rates and reinjury.

## Supplementary information

**Additional file 1.** Search

## Data Availability

The datasets used and/or analysed during the current study are available from the corresponding author on reasonable request.

## Abbreviations

ACL: Anterior cruciate ligament
RTS: Return to sport
PRISMA-ScR: Preferred Reporting Items for Systematic Reviews and Meta-Analysis Extension for Scoping Reviews
RCT: Randomised controlled trial
BPTB: Bone patella tendon bone
OM: Outcome measure
KOS-ADL: Knee Outcome Score–Activities of Daily Living
SIRAS: Sport Injury Rehabilitation Adherence Scale
