# Supplementary material for: The Influence, Barriers to and Facilitators of Anterior Cruciate Ligament Rehabilitation Adherence and Participation: a Scoping Review
Source: Sports Med Open. 2020 Jul 17;6:32. doi: 10.1186/s40798-020-00258-7 (PMC7367990; doi:10.1186/s40798-020-00258-7)
Supplement: Supplementary file 1 — Additional file 1. Search [file 40798_2020_258_MOESM1_ESM.docx]

**Supplementary File 1 – Search Terms**

**Pubmed**

(((“Anterior cruciate ligament” [tiab] OR ACL [tiab] OR "Anterior Cruciate Ligament"[Mesh])

AND

(Reconstruct* [tiab] OR "Reconstructive Surgical Procedures"[Mesh])) OR "Anterior Cruciate Ligament Reconstruction"[Mesh])

AND

(rehabilitat* [tiab] OR “exercise therapy” [tiab] OR “physical therapy” [tiab] OR physiotherapy [tiab] OR “post operative” [tiab] OR “home exercise program” [tiab] OR strength training [tiab] OR resistance training [tiab] OR weight training [tiab] OR "Rehabilitation"[Mesh] OR "Exercise Therapy"[Mesh] OR "Physical Therapy Modalities"[Mesh] OR "Resistance Training"[Mesh])

AND

(predict* [tiab] OR prevent* [tiab] OR return to sport [tiab] OR RTS [tiab] OR complian* [tiab] OR adherence [tiab] OR barrier* [tiab] OR impediment* [tiab] OR obstacle* [tiab] OR difficult* [tiab] OR problem* [tiab] OR success* [tiab] OR supervis* [tiab] OR unsupervis* [tiab] OR home [tiab] OR “group based” [tiab] OR “group training” [tiab] OR “group program” [tiab] OR “exercise group” [tiab] OR “group exercise” [tiab] OR “group exercises” [tiab] OR “exercise groups” [tiab] OR “rehabilitation group” [tiab] OR telehealth [tiab] OR digital [tiab] OR web [tiab] OR internet [tiab] OR tele-health [tiab] OR "Patient Compliance"[Mesh] OR "Treatment Adherence and Compliance"[Mesh])

**Embase**

((("Anterior cruciate ligament":ti,ab OR ACL:ti,ab OR 'Anterior Cruciate Ligament'/exp)
AND
(Reconstruct*:ti,ab OR 'Reconstructive Surgery'/exp)) OR 'Anterior Cruciate Ligament Reconstruction'/exp)
AND
(rehabilitat*:ti,ab OR "exercise therapy":ti,ab OR "physical therapy":ti,ab OR physiotherapy:ti,ab OR "post operative":ti,ab OR "home exercise program":ti,ab OR "strength training":ti,ab OR "resistance training":ti,ab OR "weight training":ti,ab OR 'Rehabilitation'/exp OR 'kinesiotherapy'/exp OR 'Physiotherapy'/exp OR 'Resistance Training'/exp)
AND
(predict*:ti,ab OR prevent*:ti,ab OR "return to sport":ti,ab OR RTS:ti,ab OR complian*:ti,ab OR barrier*:ti,ab OR impediment*:ti,ab OR obstacle*:ti,ab OR difficult*:ti,ab OR problem*:ti,ab OR success*:ti,ab OR supervis*:ti,ab OR unsupervis*:ti,ab OR home:ti,ab OR "group based":ti,ab OR "group training":ti,ab OR "group program":ti,ab OR "exercise group":ti,ab OR "group exercise":ti,ab OR "group exercises":ti,ab OR "exercise groups":ti,ab OR "rehabilitation group":ti,ab OR telehealth:ti,ab OR digital:ti,ab OR web:ti,ab OR internet:ti,ab OR tele-health:ti,ab OR 'Patient Compliance'/exp)

**CINAHL**

((((TI "Anterior cruciate ligament" OR AB "Anterior cruciate ligament" OR TI ACL OR AB ACL OR (MH "Anterior Cruciate Ligament+")))
AND
((TI Reconstruct* OR AB Reconstruct* OR (MH "Surgery, Reconstructive+")))OR (MH "Anterior Cruciate Ligament Reconstruction+")))
AND
((TI rehabilitat* OR AB rehabilitat* OR TI "exercise therapy" OR AB "exercise therapy" OR TI "physical therapy" OR AB "physical therapy" OR TI physiotherapy OR AB physiotherapy OR TI "post operative" OR AB "post operative" OR TI "home exercise program" OR AB "home exercise program" OR TI "strength training" OR AB "strength training" OR TI "resistance training" OR AB "resistance training" OR TI "weight training" OR AB "weight training" OR (MH "Rehabilitation+") OR (MH "Therapeutic Exercise+") OR (MH "Physical Therapy+") OR (MH "Resistance Training+")))
AND
((TI predict* OR AB predict* OR TI prevent* OR AB prevent* OR TI "return to sport" OR AB "return to sport" OR TI RTS OR AB RTS OR TI complian* OR AB complian* OR TI adherence OR AB adherence OR TI barrier* OR AB barrier* OR TI impediment* OR AB impediment* OR TI obstacle* OR AB obstacle* OR TI difficult* OR AB difficult* OR TI problem* OR AB problem* OR TI success* OR AB success* OR TI supervis* OR AB supervis* OR TI unsupervis* OR AB unsupervis* OR TI home OR AB home OR TI "group based" OR AB "group based" OR TI "group training" OR AB "group training" OR TI "group program" OR AB "group program" OR TI "exercise group" OR AB "exercise group" OR TI "group exercise" OR AB "group exercise" OR TI "group exercises" OR AB "group exercises" OR TI "exercise groups" OR AB "exercise groups" OR TI "rehabilitation group" OR AB "rehabilitation group" OR TI telehealth OR AB telehealth OR TI digital OR AB digital OR TI web OR AB web OR TI internet OR AB internet OR TI tele-health OR AB tele-health OR (MH "Patient Compliance+")))

**SportDiscus**

((((TI "Anterior cruciate ligament" OR AB "Anterior cruciate ligament" OR TI ACL OR AB ACL OR (DE "ANTERIOR cruciate ligament")))
AND
((TI Reconstruct* OR AB Reconstruct* OR (MH "Surgery, Reconstructive+")))OR (DE "ANTERIOR cruciate ligament surgery")))
AND
((TI rehabilitat* OR AB rehabilitat* OR TI "exercise therapy" OR AB "exercise therapy" OR TI "physical therapy" OR AB "physical therapy" OR TI physiotherapy OR AB physiotherapy OR TI "post operative" OR AB "post operative" OR TI "home exercise program" OR AB "home exercise program" OR TI "strength training" OR AB "strength training" OR TI "resistance training" OR AB "resistance training" OR TI "weight training" OR AB "weight training" OR (DE "REHABILITATION") OR (DE "EXERCISE therapy") OR (DE "PHYSICAL therapy practice") OR (DE "WEIGHT training injuries")))
AND
((TI predict* OR AB predict* OR TI prevent* OR AB prevent* OR TI "return to sport" OR AB "return to sport" OR TI RTS OR AB RTS OR TI complian* OR AB complian* OR TI adherence OR AB adherence OR TI barrier* OR AB barrier* OR TI impediment* OR AB impediment* OR TI obstacle* OR AB obstacle* OR TI difficult* OR AB difficult* OR TI problem* OR AB problem* OR TI success* OR AB success* OR TI supervis* OR AB supervis* OR TI unsupervis* OR AB unsupervis* OR TI home OR AB home OR TI "group based" OR AB "group based" OR TI "group training" OR AB "group training" OR TI "group program" OR AB "group program" OR TI "exercise group" OR AB "exercise group" OR TI "group exercise" OR AB "group exercise" OR TI "group exercises" OR AB "group exercises" OR TI "exercise groups" OR AB "exercise groups" OR TI "rehabilitation group" OR AB "rehabilitation group" OR TI telehealth OR AB telehealth OR TI digital OR AB digital OR TI web OR AB web OR TI internet OR AB internet OR TI tele-health OR AB tele-health OR (DE "PATIENT decision making")))

**Web of Science**

(((("Anterior cruciate ligament" OR ACL OR "Anterior Cruciate Ligament"))
AND
((Reconstruct* OR "Reconstructive Surgical Procedures"))OR "Anterior Cruciate Ligament Reconstruction"))
AND
((rehabilitat* OR "exercise therapy" OR "physical therapy" OR physiotherapy OR "post operative" OR "home exercise program" OR "strength training" OR "resistance training" OR "weight training" OR Rehabilitation OR "Exercise Therapy" OR "Physical Therapy Modalities" OR "Resistance Training"))
AND
((predict* OR prevent* OR "return to sport" OR RTS OR complian* OR adherence OR barrier* OR impediment* OR obstacle* OR difficult* OR problem* OR success* OR supervis* OR unsupervis* OR home OR "group based" OR "group training" OR "group program" OR "exercise group" OR "group exercise" OR "group exercises" OR "exercise groups" OR "rehabilitation group" OR telehealth OR digital OR web OR internet OR tele-health OR "Treatment Adherence and Compliance"))
